# Potential Impact of Oral Inflammations on Cardiac Functions and Atrial Fibrillation

**DOI:** 10.3390/biom8030066

**Published:** 2018-08-01

**Authors:** Ghazal Aarabi, Renate B. Schnabel, Guido Heydecke, Udo Seedorf

**Affiliations:** 1 Department of Prosthetic Dentistry, Center for Dental and Oral Medicine, University Medical Center Hamburg-Eppendorf, 20246 Hamburg, Germany; g.aarabi@uke.de (G.A.); g.heydecke@uke.d (G.H.); 2 Department of General and Interventional Cardiology, University Heart Center Hamburg-Eppendorf, 20251 Hamburg, Germany; r.schnabel@uke.de; 3 DZHK (German Center for Cardiovascular Research), Partner Site Hamburg/Kiel/Lübeck, 20251 Hamburg, Germany

**Keywords:** oral health, atrial fibrillation, bacteremia, autoimmunity, autonomous nervous system, bacterial toxins

## Abstract

Inflammation may be a risk factor for atrial fibrillation (AF). Oral infections frequently lead to chronic inflammation, such as gingivitis, periodontitis, and endodontic lesions. In this narrative review, we consider five basic pathogenic mechanisms that involve oral infections and inflammations in the pathogenesis of AF: (1) low level bacteremia by which oral bacteria enter the blood stream at inflamed sites of the oral cavity and invade the heart; (2) Systemic inflammation induced by inflammatory mediators, which are released from the sites of oral inflammation into the blood stream, affecting cardiac remodeling; (3) autoimmunity against molecular structures expressed in the heart caused by the host immune response to specific components of oral pathogens; (4) potentially arrhythmic effects mediated by activation of the autonomous nervous system triggered by oral inflammations; and (5) arrhythmic effects resulting from specific bacterial toxins that are produced by oral pathogenic bacteria. A number of studies support the involvement of all five mechanisms, suggesting a potentially complex contribution of oral inflammations to the pathogenesis of AF.

## 1. Introduction

Atrial fibrillation (AF) is the most common persistent cardiac arrhythmia occurring in clinical practice. In Europe, around 6 million people suffer from AF; in the entire western world 1.5–2% of the population is affected, with more men than women. The prevalence of AF is age-dependent, rising from <0.5% in 40–50-year-old individuals to 5–15% in 80-year-olds and will continue to increase over the next 50 years [1,2]. After reaching the age of 40, 25% of men and women are at risk of developing AF during their later life [3]. Around 70% of affected patients are between 65 and 85 years old [4]. Atrial fibrillation is a risk factor for heart failure, dementia, and stroke [5,6,7].

Risk factors for AF include age, gender, high body mass index, hypertension, cardiovascular disease, valvular disease, and heart failure among others [3]. However, these conventional risk factors explain only a minor fraction of all AF cases, suggesting that additional risk factors may be relevant. Among these additional risk factors, a substantial heritable component is of note [2]. Large twin studies estimated the total heritability of AF to be as high as 62% [8] and recent genome-wide association studies led to the identification of risk variants at over 100 genetic loci [9], many of which located at genes implicated in heart defects, ion channels, and heart muscle structure and function. In addition to these genetic markers, there is a contribution of inflammation to AF risk that is likely not sufficiently covered by the existing risk models [10]. That inflammation may contribute to AF is supported by a number of clinical observations: (i) AF is associated with pericarditis [11]; (ii) the incidence of post-operative AF is influenced by IL-6 genotype and level [12]; and (iii) the observation that 25% to 40% of people develop AF after cardiac surgery [13]. It could be demonstrated that the time course of AF after cardiac surgery coincides with the activation of the complement system and the release of pro-inflammatory cytokines [13,14].

Humans are affected by a great number of infections and chronic inflammations. Over a lifetime, oral infections are important, because of their longstanding nature and high frequency in the population. Infective endocarditis, for instance, which is caused by bacteria that colonizes the teeth, often results from bacteremia after toothbrushing [15]. Participants with high plaque and calculus scores were at a 3.78- and 4.43-fold increased risk of developing bacteremia and bleeding induced by toothbrushing was associated with an almost eight-fold increased risk for bacteremia [16]. In a recent study, 14 of 22 cases of pericarditis were positive for endodontitis-related bacteria (63.6%), while eight were positive for periodontis-related bacteria (36.4%) [17].

It appears conceivable that cardiac arrhythmias could potentially also be affected by the systemic inflammation, which is known to accompany oral inflammations and/or by autoimmunity against molecular structures expressed in the heart caused by the host immune response to specific oral pathogens. Finally, arrhythmic effects resulting from activation of the autonomic nervous system and from specific bacterial toxins that are produced by oral pathogenic bacteria could also play a role. To evaluate these potential mechanisms, we screened the published literature and reviewed the most important findings in this context. 

## 2. Frequent Forms of Chronic Oral Infections Potentially Associated with Atrial Fibrillation

**Figure 1:**
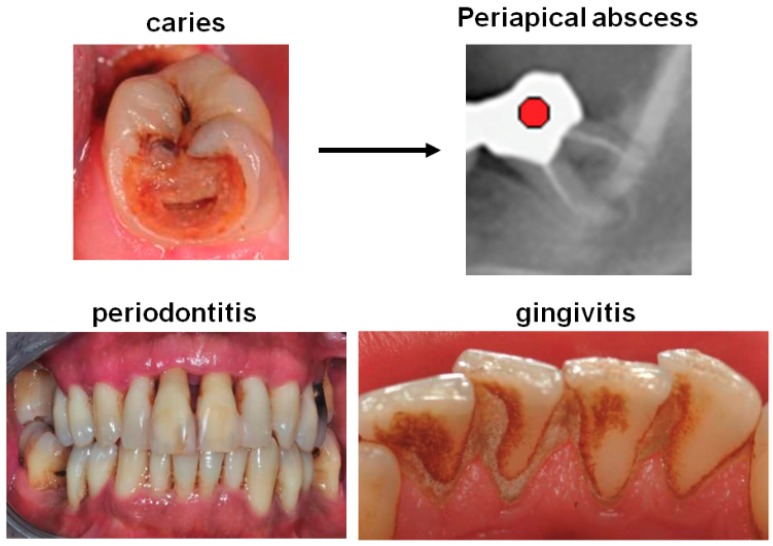
Oral inflammation affecting the teeth, gingiva, and the periodontium. Shown are examples of a tooth affected by severe caries, a tooth with abscess formation revealed by orthopantomography, a denture with severe periodontitis with evidence of extensive loss of attachment and gingival recession at most teeth, and an example of gingivitis with extensive calculus formation and a swollen and reddened inflamed gingiva.

Oral infections potentially associated with AF include infections of the teeth, such as caries, which may lead to endodontic lesions, and infections of the gingival tissues surrounding the teeth, such as gingivitis and periodontitis, the latter affecting the tooth-supporting structures [18] (see Figure 1 for examples). Gingivitis develops when bacteria, which form a biofilm at the tooth surface, infect the surrounding gingiva and trigger an immune response, resulting in swelling, redness and bleeding of the gingiva [19]. The inflammation can progress to periodontitis, if the bacteria and the accompanying inflammation migrate along the root surface and penetrate into the tooth supporting structures [20]. In Europe, almost 50% of over 30 years old individuals are affected by some form of periodontitis and over 10% have severe chronic periodontitis [21]. In most populations, 5–20% are affected by severe, generalized periodontitis [22,23]. Endodontic inflammations result mostly from deep dental caries, when the infection penetrates through the teeth’s root canal to the apex of the teeth’s root where a periapical abscess is formed [24]. Thirty to sixty percent of middle-aged Scandinavians [25,26] and Canadians [27] were shown to be affected by at least one periapical abscess, suggesting that a large fraction of the population is exposed to this kind of inflammation.

## 3. Potential Role of Bacteremia

The oral microbiome, which is composed of more than 700 different bacterial species, populate all oral hard and soft surfaces [19]. To prevent bacteria from entering the body, gingival epithelial cells provide a mechanical barrier and release antimicrobial peptides (i.e., hBD-2, hBD-3, cathelicidin LL-37). If bacteria still invade the tissue, immune cells and monocytes, which can release a wide range of pro-inflammatory mediators, are attracted by chemo-attractans, such as interleukins 1 and 8 (IL-1 and IL-8) [28,29,30,31]. The edematous gingival and periodontal tissues are prone to bleeding, which facilitates the penetration of oral bacteria into the bloodstream. In addition, the bacteria can enter the body after internalization, together with phagocytic immune cells [32,33]. Transient bacteremias were demonstrated to occur in patients with periodontitis after tooth brushing and following periodontal treatment [33,34,35]. Oral bacteria and/or their DNA have been detected in human atherosclerotic lesions, the pericardial fluid, heart valves, and thrombi in many studies [17,36,37,38,39,40]. A recent meta-analysis of 63 studies that included 1791 patients confirmed the presence of 23 oral bacterial species in atherosclerotic plaques [41]. *Campylobacter rectus*, *Porphyromonas gingivalis*, *Porphyromonas endodontalis*, *Prevotella intermedia*, and *Prevotella nigrescens* were only detected in cardiac tissue, whereas the other species showed a more widespread distribution. Bacteria from endodontic lesions, such as *Streptococcus mutans*, could be detected in biopsies from heart valves (40% positive) and atheromas (48% positive) [42]. The polymerase chain reaction (PCR) signals for this bacterium were stronger than those of bacterial species related to periodontitis. Collectively, these results provide convincing evidence for the ability of a wide range of oral bacteria to invade the body most likely via the blood stream. However, evidence for their presence within the myocardium and their association with the cardiac conduction system is still lacking.

## 4. Potential Role of Systemic Inflammation

Higher C-reactive protein (CRP), which is a sensitive biomarker for systemic inflammation, was associated with AF in the Cardiovascular Health Study. More individuals in the fourth CRP quartile had AF than in the first quartile (7.4% vs. 3.7%, adjusted odds ratio (OR) 1.8, 95% confidence interval (CI) 1.2 to 2.5; *p* = 0.002) and baseline CRP level predicted the risk for developing future AF (fourth vs. first quartile adjusted hazard ratio 1.31, 95% CI 1.08 to 1.58; *p* = 0.005), indicating that CRP is not only associated with the presence of AF, but also predicts patients at increased risk for future development of AF [10].

It is well known that oral infections elevate systemic CRP levels consistently [43,44,45,46,47,48]. The highest levels have been observed in patients with acute and chronic endodontic lesions (alveolar abscesses) [49,50]. In addition to CRP, oral inflammation affects the circulating levels of many other inflammatory markers and cytokines (see Table 1 for details) [50,51]. The pro-inflammatory mediator IL-6 stimulates the production of CRP and fibrinogen by the liver, resulting in an acute-phase reaction that has pro-inflammatory and pro-atherogenic effects [46]. Clinical studies have shown that circulating IL-6 is not only elevated in patients with periodontitis, but also in patients with heart failure—a major risk factor for AF (see review by Wollert and Drexler [52]). Interleukin-6 stimulates cardiomyocyte hypertrophy and apoptosis and may contribute to fibrosis during heart failure thereby altering cardiac conduction and contributing to AF. In renal failure patients, elevated circulating IL-6 was identified as a risk factor for AF [53] and increased circulating IL-6 levels have been demonstrated in coronary heart disease CHD patients with AF [54]. Circulating IL-6 also correlated with the extent of left ventricular hypertrophy of the heart, which is an important risk factor for AF, in a large group of 971 patients [55]. Finally, it could be shown that polymorphisms in the promoter of the IL-6 gene, which influence the concentration of circulating IL-6, were associated with the risk of post-operative AF [12,56]. In conclusion, the published data suggest that IL-6 may favor AF due to its direct effects on electrophysiological remodeling of the heart.

In contrast to IL-6, there is little evidence in the literature for a direct effect on cardiac electrophysiological remodeling for CRP [57]. Although circulating CRP is associated with AF [58], it cannot be ruled out that it is merely a bystander of cardiac remodeling involved in disease progression. Marott et al. were able to show that four CRP gene variants, which, in total, were associated with a 63% increase in circulating CRP, did not contribute to an increase in AF risk [59]. However, elevated CRP may predispose patients to persistence of AF via triggering arrhythmogenic foci, which worsen the arrhythmia over time and lead to worsened outcomes [10]. High CRP could reflect ongoing ventricular remodeling, as was also suggested previously for acute coronary syndromes, in which high CRP was associated with worsened mortality and left ventricular dysfunction [60]. The proposed link between inflammation and AF has potential therapeutic implications, because there are therapeutic strategies available, which target systemic inflammation (i.e., statins, canakinumab [61,62]). A recent systematic review of controlled trials with statins, which included six studies comprising 3557 patients in sinus rhythm, showed that statins led to a decreased risk of AF compared to controls ((OR) 0.39, 95% CI 0.18 to 0.85, *p* = 0.02). Statins appeared to be more effective if used in secondary prevention of AF (OR 0.33, 95% CI 0.10 to 1.03, *p* = 0.06) than in new-onset or postoperative AF (OR 0.60, 95% CI 0.27 to 1.37, *p* = 0.23) [63]. However, it could not be differentiated to what degree the potential benefit of statin treatment in patients with AF were due to lowering low density lipoprotein (LDL) cholesterol and inhibiting coronary artery disease (CAD) progression as opposed to their anti-inflammatory effects. Statins were also demonstrated to have beneficial effects on the periodontal status in hyperlipidemic patients, which were interpreted to be due to their anti-inflammatory effects [64,65].

## 5. Potential Role of Autoimmunity in Atrial Fibrillation

Atrial fibrillation occurring at young age has been observed in several autoantibody-associated diseases, such as rheumatoid arthritis, systemic lupus erythematosus, and antiphospholipid syndrome [66,67,68]. Among the many self-antigens that have been proposed as potential targets of the self-directed immune responses to the heart [69], heat shock proteins (HSPs) are of special interest, because changes in cardiac HSP60/65 expression have been observed in patients with AF [70,71] and auto-reactivity to HSPs has been observed patients with periodontal disease [72].

Heat shock proteins are ubiquitous molecular chaperones functioning in cellular stress protection, which are evolutionary highly conserved [73]. *Porphyromonas gingivalis* and many other bacteria involved in oral infections, express homologs to human HSPs [74]. The HSP60/65 homolog of *Porphyromonas gingivalis* (called GroEL) has the ability to induce a humoral and cellular immune response in humans which is cross-reacting with the endogenous HSPs expressed by the host [75]. An association between antibodies to HSP60/65 and the occurrence of postoperative AF was reported, suggesting a potential involvement of HSP60/65 antibodies in the pathogenesis of AF. It was postulated that individuals with high preoperative levels of HSP60/65 antibodies could develop a harmful autoimmune reaction under the stressful operative conditions leading to induction of HSP expression and presence of HSPs on the surface of cardiomyocytes [76], which could subsequently cause myocyte injury and finally AF.

Activation of the HSP autoimmunity mechanism has been firmly established to operate in patients with periodontitis infected with *Porphyromonas gingivalis* [77,78,79,80]. Humoral and cellular immunity against HSPs is thought to be a normal feature of healthy humans, which participates in the protection against microbial infections [81]. The ability of the immune system to induce this potentially dangerous immune response depends on the strength of the HSP60/65 immunoresponse, which is elevated in patients with periodontitis. In addition, interaction with highly polymorphic major histocompatibility complex (MHC) class I and II epitopes on the cells’ surface is required to trigger the response [82].

## 6. Potential Role of the Autonomic Nervous System

**Figure 2:**
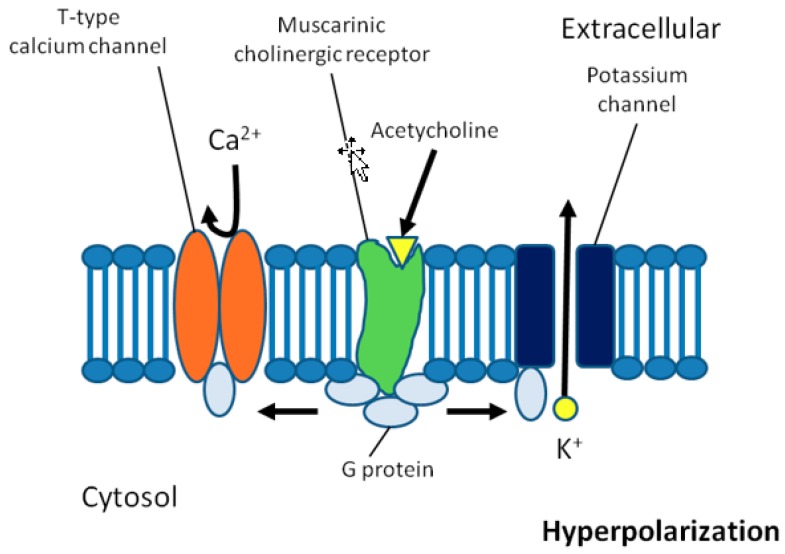
Potential role of the autonomous nervous system in the pathophysiology of atrial fibrillation. The cholinergic anti-inflammatory pathway, which is mediated by the parasympathetic nervous system via the vagus nerve, leads to secretion of acetylcholine, which binds to muscarinic cholinergic receptors. The receptors open potassium channels through stimulatory G proteins and close funny channels and T-type calcium channels through an inhibitory G protein. This leads to hyperpolarization, shorter duration of action potentials, a shortened effective refractory period, and a higher susceptibility to reentry at sites where acetylcholine is released in the atrium. The atria are only sparsely innervated by the sympathetic nervous system, which may also be activated during inflammation.

The mechanisms described above link AF to chronic inflammation, leading to a structurally damaged heart that is prone to developing AF over time. However, AF can also occur spontaneously in people with a structurally healthy heart, mostly in the form of paroxysmal AF (PAF), which is characterized by episodes of intermittent AF occurring commonly in younger people. Psychic stress and infections were among the most common triggering factors of AF episodes identified in a Swedish survey of hospitalized patients with PAF [83]. One of the many responses of the body to an infection is activation of the autonomic nervous system. An important functional component of this system is the inflammatory reflex, a sensory pathway to detect and localize the presence of inflammation by the body’s autonomic nervous system [84]. Afferent signals are transmitted from the inflamed tissue (i.e., the atrium) to the brain via the vagus nerve, which activates the cholinergic anti-inflammatory pathway by triggering efferent vagus nerve signaling leading to secretion of acetylcholine (ACh) locally in the vicinity of macrophages at the inflamed sites of the tissue [84]. Acetylcholine binds to nicotinic Ach receptors that are expressed by macrophages and induces macrophage deactivation to limit the release of pro-inflammatory cytokines. In the heart, ACh can additionally activate Ach-sensitive potassium channels, which shortens the duration of action potentials and the effective refractory period locally at sites where Ach is released in the atrium, thereby increasing the susceptibility to reentry, which may contribute to induction and maintenance of AF [85]. The proposed mechanism is illustrated in Figure 2.

In addition to the cholinergic anti-inflammatory pathway, which is a component of the parasympathetic nervous system, inflammation is also regulated in a complex manner by its antagonist, the sympathetic nervous system [86]. Its activation leads, among others, to the release of catecholamines, such as norepinephrine and epinephrine, at the inflamed sites that can be pro- or anti-inflammatory depending on the cellular and topological context (for details see [86]). States of adrenergic stimulation, such as during exercise, were shown to be triggers of AF in some patients [87] and increased atrial sympathetic innervations were observed in patients with persistent AF [88]. Moreover, ectopic beats arising from the pulmonary veins, which are a frequent cause of AF, can be inhibited by ablation of the relevant autonomic foci, β-adrenergic receptor blockers, sodium channel blockers, and calcium channel blockers [89].

The heart has an intrinsic autonomic nervous system with autonomic ganglia, which are mainly located in the epicardial fat near the pulmonary vein-atrial junction and the ligament of Marshall, regions that are also densely innervated by the body’s autonomic nervous system [90]. This topological situation might be of relevance for the interaction between the body’s autonomic and intrinsic cardiac systems. It could be demonstrated that catheter ablation of these regions could improve outcomes in some AF patients compared to pulmonary vein isolation alone [91]. Episodes of AF are triggered in some patients when sympathetic activity is followed by an abrupt change to vagal dominance [92]. An initial increase in sympathetic tone followed by a marked shift towards vagal dominance was also observed before the onset of AF in some patients with ectopy arising from the pulmonary veins [93]. Thus, shifting and/or simultaneous activation of sympathetic and parasympathetic signaling, which is typical during states of inflammation, may be able to provoke AF in susceptible people.

Severe bacteremia, such as that occurring during sepsis, is a very effective inducer of the autonomic nervous system and can trigger AF in humans [94,95,96]. However, whether or not the more moderate forms of bacteremia, which are typically occurring during oral inflammation and dental surgery, can trigger the autonomic nervous system strongly enough to induce AF is currently unclear. Although, periodontitis was an independent predictor of arrhythmic events in patients with AF and arrhythmias occurred in a very large fraction (93.6%) of hospitalized AF patients with severe periodontitis [97], the contribution of the autonomic nervous system was not addressed in this study.

## 7. Potential Role of Bacterial Toxins

Patients affected by oral infections are exposed to many toxins, metabolic products, and proteins of bacterial origin, which may influence the myocardium. Lipopolysaccharide (LPS), for instance, which is an endotoxin that is produced by Gram-negative bacteria, down-regulates the expression of L-type calcium channels and shortens the effective refractory period in a rat model of AF, which is proposed as a mechanism underlying sepsis-induced AF [98]. Streptolysin O, a streptococcal toxin, was shown to be involved in cardiomyocyte contractile dysfunction, by impairing the response of cardiac cells to electrical pacing by inducing influx of calcium into the cytosol through toxin-mediated pores in the plasma membrane [98]. Results from experiments performed with isolated guinea pig hearts perfused with streptolysin O were most consistent with a defect in the atrioventricular conduction system and atrial release of ACh [99].

*Aggregatibacter actinomycetemcomitans* (a Gram-negative, facultative anaerobe bacterium associated with localized aggressive periodontitis) was recently shown to secrete 179 different proteins [100], and various strains of *Porphyromonas gingivalis* were shown to secrete up to 250 proteins [101], some of which may affect cardiac remodeling and induce AF. One of these proteins is *P. gingivalis* peptidylarginine deiminase (PPAD), an enzyme involved in protein citrullination. Protein citrullination is a post-translational modification by which l-arginine is enzymatically converted to l-citrulline [102,103]. Humans have five peptidylarginine deiminase (PAD) isoenzymes, which fulfill important physiological roles during inflammation, apoptosis, embryonic development, and epigenetic gene regulation [103,104]. Healthy humans are generally immune tolerant to citrullinated proteins. However, since *P. gingivalis* PAD citrullinates not only human proteins (i.e., α-enolase and vimentin), but also some of its own proteins, it was hypothesized that the long-lasting exposure to highly citrullinated bacterial and host proteins during periodontitis may trigger breakdown of immune tolerance to citrullinated epitopes in susceptible individuals, thereby favoring autoimmunity and the development of rheumatoid arthritis [104,105,106,107]. In line with this hypothesis, periodontitis was independently associated with rheumatoid arthritis in multiple epidemiological studies (reviewed in ref. [108]).

Although *Aggregatibacter actinomycetemcomitans* has no PAD encoding gene and does not secrete PAD, changes in citrullination were also observed to occur in infections caused by this periodontal pathogen, which induced hypercitrullination of a large number of proteins in host neutrophils [109]. The elucidated mechanism involves the pore-forming toxin leukotoxin A (LtxA), which triggers dysregulated activation of host PADs and export of the hypercitrullinated proteins from neutrophils, which act as citrullinated autoantigenes, favoring the formation of anticitrullinated protein antibodies (ACPA) and rheumatoid arthritis. The results suggest that infections with some of the many pathogenic bacteria, which secrete pore-forming toxins with similar properties as LtxA, may have the more general ability to induce hypercitrullination and to trigger formation of ACPA in humans.

Hypercitrullination of proteins in cardiomyocytes of the failing heart is likely important, because rheumatoid arthritis is strongly associated with AF [66]. Anticitrullinated protein antibodies were significantly associated with altered left ventricular structure and function in a recent study on patients with rheumatoid arthritis [110], and citrullinated proteins with impaired function could be identified in human myocardial samples from healthy and heart failure patients [111]. However, specific effects that support an ability of LtxA to trigger AF via citrullination or other mechanisms have not been reported.

## 8. Discussion

**Figure 3:**
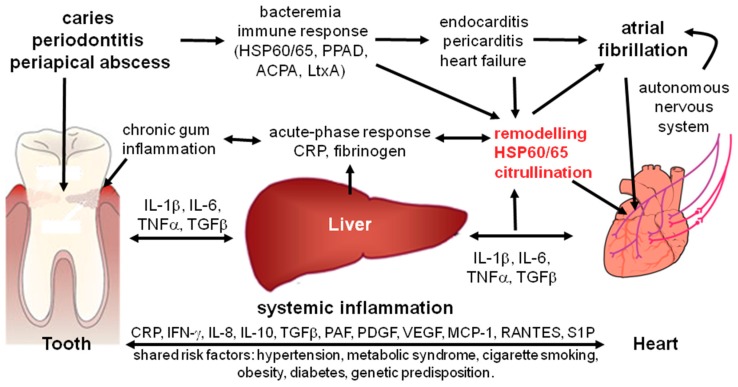
Potential role of the oral inflammation in the pathogenesis of atrial fibrillation. Chronic oral inflammation induced by bacterial biofilm (gingivitis, periodontitis, and apical endodontic lesions) cause a localized inflammation, which affects cardiac structural and electrophysiological remodeling linked to atrial fibrillation by: (i) low level bacteremia by which oral bacteria enter the blood stream at inflamed sites of the oral cavity and invade the heart; (ii) systemic inflammation induced by inflammatory mediators which are released from the sites of oral inflammation into the blood stream, affecting ventricular remodeling; (iii) autoimmunity against molecular structures expressed in the heart, such as HSP60/65 and citrullinated cardiac proteins, caused by the host immune response to specific components of oral pathogens; (iv) arrhythmogenic effects mediated by activation of the autonomic nervous system during inflammation; (v) effects resulting from specific bacterial proteins and toxins, such *Porphyromonas gingivalis* PAP and leukotoxin A (LtxA) that are produced by oral pathogenic bacteria and induce the formation of anticitrullinated protein antibodies (ACPA). Abbreviations: CRP: C-reactive protein; IL: interleukin; IFN: interferon; MCP: monocyte chemoattractant protein; PAF: platelet activating factor; PDGF: platelet-derived growth factor; RANTES: regulated on activation, normal T cell expressed and secreted; S1P: sphingosine-1-phosphate; TNF: tumor necrosis factor; VEGF: vascular endothelial growth factor.

The presented findings support that chronic oral inflammations likely affect the pathogenesis of AF by multiple pathways, suggesting the existence of an oral-heart-axis (schematically illustrated in Figure 3). Although none of the described mechanisms is specific for oral inflammations, due to the high prevalence and chronic nature of oral inflammations, they may have a profound public health impact. Recently, over 100 genetic loci could be identified as functional candidates involved in AF [112]. Among these genes, a minority, such as IL6, IL19, TGFB1, CARD8, has functions in infection and inflammation, whereas the majority is likely involved in cardiac and skeletal muscle function and integrity, cardiac embryonic development, intracellular calcium handling, angiogenesis, and cardiac ion channeling [112]. Based on the findings described in this review, one might hypothesize that interaction between the genetic predisposition, which seems to mostly modulate the activity of pathways involved in structural and functional cardiac aspects, with chronic inflammation may be crucial for the emergence of the AF-prone heart.

Activation of the HSP60 autoimmunity mechanism has been firmly established to operate in patients with periodontitis infected with *Porphyromonas gingivalis*. However, although it has been demonstrated that the relevant HSPs are induced in the stressed heart, the precise consequences on cardiac outcomes and the quantitative relevance of the HSP related autoimmune response have not been sufficiently addressed. Whether the HSPs of endodontic pathogens elicit a similar immune response as was demonstrated for *Porphyromonas gingivalis* GroEL, is unknown. The studies concerning the roles of citrullination and ACPA in the pathogenesis of various forms of heart failure and AF are still at the very beginning. Thus far, the studies have been restricted to patients with rheumatoid arthritis, which may be considered to represent an extreme. Since the presence of ACPA in the circulation precedes the onset of rheumatoid arthritis up to nine years [113], it would be interesting to investigate ACPA in less extreme cases without rheumatoid arthritis, but with heart failure or AF.

If bacteremia occurring during oral inflammations and dental procedures, such as tooth extractions, can provide a strong enough stimulus for the autonomic nervous system to trigger AF it should be addressed in further clinical studies.

Interesting results have recently led to the identification of a vast number of factors that are secreted by oral pathogens (secretome). These factors warrant further characterization with respect to their arrhythmogenic effects.

## Figures and Tables

**Table 1:** Cytokines Linked to Oral Inflammations

Cytokine	Function
IL-8, MIP-1, MCP-1, RANTES	Chemotactic
IL-1α, IL-1β, TNFα, IL-6, PAF	Pro-inflammatory
IL-1RA, IL-4, IL-10	Anti-inflammatory
IFN-γ, IL-2, IL-4, IL-5, IL-7	Immunoregulatory
PDGF, EGF, FGF, IGF, VEGF	Growth factor

EGF, epidermal growth factor; FGF, fibroblast growth factor; IFN, interferon; IGF insulin-like growth factor; IL, interleukin; IL-1RA, interleukin-1-receptor antagonist; MIP, macrophage inflammatory protein; MCP, monocyte chemotactic protein; PAF, platelet activating factor; PDGF, platelet derived growth factor; RANTES, regulated upon activation, normal T cell expressed and secreted; VEGF, vascular endothelial growth factor.
